# Development and validation of a DNA repair gene signature for prognosis prediction in Colon Cancer

**DOI:** 10.7150/jca.46328

**Published:** 2020-08-12

**Authors:** Xin Wang, Cong Tan, Min Ye, Xu Wang, Weiwei Weng, Meng Zhang, Shujuan Ni, Lei Wang, Dan Huang, Zhaohui Huang, Midie Xu, Weiqi Sheng

**Affiliations:** 1 Department of Pathology, Fudan University Shanghai Cancer Center, Shanghai, 200032, China.; 2 Department of Oncology, Shanghai Medical College, Fudan University, Shanghai, 200032, China.; 3 Institute of Pathology, Fudan University, Shanghai 200032, China.; 4 Wuxi Cancer Institute, Affiliated Hospital of Jiangnan University, Wuxi, Jiangsu, China.

**Keywords:** colon cancer, DNA repair, prognosis, TCGA, GEO

## Abstract

Aberrant expression of DNA repair genes (DRGs) can be related to tumor progression and clinical outcomes in colon cancer. Here, we aimed to establish a DRGs signature to identify the vital prognostic DRGs in colon cancer. Firstly, gene set enrichment analysis (GSEA) was performed to demonstrate the association between abnormal expression level of DRGs and tumorigenesis. Then, a total of 476 DRGs were obtained for detecting candidate biomarkers in randomly selected 295 cases from The Cancer Genome Atlas (TCGA) colon cancer cohort. Eleven genes were screened by LASSO Cox regression analyses to develop the prognostic model. Then, the prognostic model and the expression levels of the eleven genes were validated using the internal validation dataset (the rest 125 cases in TCGA cohort) and an external validation dataset (obtained from Gene Expression Omnibus dataset). Further analysis revealed the independent prognostic capacity of the prognostic model in relation to other clinical characteristics. The receiver operating characteristic (ROC) curve analysis confirmed the good performance of the prognostic model. Furthermore, we provided a nomogram for interpreting the clinical application of the 11-DRG signature. In conclusion, we propose a newly developed 11-DRG signature as a practical prognostic predictor for patients with colon cancer, which can facilitate the individualized counselling and treatment.

## Introduction

Colon cancer is one of the most common and lethal malignancies with an estimated 1,096,601 new cases and 551,269 deaths reported in Globocan 2018 1. A large amount of patients benefits from early detection and comprehensive treatment. However, clinical outcomes of patients occasionally present unpredictable diversity even with similar treatment strategies 2, 3. Although much effort has been made to identifying pathological or biochemical markers for prognosis prognostication, results from single factor is unsatisfying. Recently, with advancement of the next generation sequencing technology, the new insight into the molecular network of cancer hallmarks and the availability of high-throughput genome and transcriptome data offer us a richer theoretical and practical basis for prognosis prediction of colon cancer 4, 5.

DNA repair occurs all the time in human cells to identify and correct damage to the DNA molecules that encode its genome 6. Complex signaling pathways, including base excision repair, nucleotide excision repair, and mismatch repair, work to deal with varieties of DNA damage from endogenous or exogenous sources. DNA damage and repair is a widely concerned hallmark in tumor biology 7. Researchers have reported the association between the aberrant expression of DNA repair genes (DRGs) with the tumor generation 8-10. Disorders in DNA repair process has been recognized as one of the critical characteristics in tumor generation and progress in molecular biological behavior in colon cancer 11, 12. In view of the important role that DRGs play in colon cancer, attentions should be paid to understand their connections with prognosis.

In this study, we aim to identify the activity of DRGs in colon cancer and establish a prognostic modal with DRGs. Firstly, we noted the differences in the transcriptional profile of epithelium cells, and predicted the actions of DRGs during the malignant transformation of colon by functional analysis. Then, we applied LASSO Cox regression analysis to establish a prognostic model for patients with colon cancer. Furthermore, we performed internal and external data validation to assess the robustness of the classifier. Finally, we analyzed the relationship between the modal and clinical information. The results demonstrated the independency of our 11-DRG signature in predicting clinical outcomes for patients with colon cancer.

## Material and Methods

### Data download and processing

Transcriptome profiling and clinical data of TCGA-colon adenocarcinoma (COAD) cohort were downloaded from the GDC Data Portal (https://portal.gdc.cancer.gov/). Only samples with complete prognostic data were included. The pathologic stages of 420 cases were reconfirmed according to the seventh edition of American Joint Committee on Cancer staging system 13. TCGA-COAD counts were normalized with R package “DEseq2”. Four colon cancer datasets, including GSE21510, GSE24514, GSE32323 and GSE39582 (Table S1), were downloaded from the Gene Expression Omnibus (GEO) database (https://www.ncbi.nlm.nih.gov/geo/). All the GEO datasets were normalized with log2 transformation and probe annotation by using “limma” package, if necessary. For DRGs identification, we choose “GO_DNA_REPAIR” gene list, containing 544 DRGs, downloaded from “GO biological process” gene set, Gene Set Enrichment Analysis (GSEA), to obtain sufficient DRGs. Finally, 476 DRGs were annotated and detected in above datasets (Table S2).

### Gene Set Enrichment Analysis (GSEA)

Samples from three GEO datasets, GSE21510, GSE24514 and GSE32323, were divided into normal colon and tumor group depended on pathologic diagnosis. Functional analysis was conducted by “GSEA” function using “clusterProfiler” package. Hallmarks gene set "h.all.v7.0.entrez.gmt" was set as the reference gene database, and cutoff criteria was set to *p* < 0.05 and *q* < 0.25. Common hallmarks of GSEA were presented in Venn diagram.

### Lasso-Cox regression modal

The Cox proportional hazards model with LASSO penalty could establish the relationship between predictor genes and survival time, and at the same time, reduce the coefficients of correlated predictors while making pick or discard decision 14. Here, we tried to establish a DRGs prognostic modal based on LASSO Cox regression, with “glmnet” package in R. A total of 476 DRGs were included for detecting candidate biomarkers. The optimal penalty parameter λ (lambda) was selected by cross-validation method, “lambda.min” was determined as the best selection, which is the values of λ that gives minimum mean cross-validated error. Finally, the modal output a risk score of each patient, which was calculated by summing relative expression levels of the vital DRGs (*Exp_i_*) and the corresponding LASSO coefficient (*L_i_*): 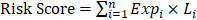.

To predict the prognosis, patients were divided into high-risk or low-risk groups, according to the risk score. The best cutoff of risk score was determined when optimal Area Under Curve (AUC) in Receiver operating characteristic (ROC) curve achieved for predicting 5-year survival in the training set, by utilizing the assessment of Youden's index 15, 16. Kaplan-Meier survival analysis was used to predict clinical outcomes for patients in each cohort. ROC curve was conducted to calculate AUC for one-year, three- year and five-year overall survival in training, test, and validation set, to estimate the accuracy of the modal.

### Statistical assessment of the modal and visualization

Boxplot was used to display the distribution of Risk Score in patient with various clinic characteristics. *T* test or one-way *ANOVA* was applied to evaluate differences between groups. Relationship between risk group of patients and clinic parameters was assessed by chi-square test or fisher exact test, as appropriate. Univariate and multivariate Cox regression of the modal was used to test independency of the modal. Nomogram and calibration analysis were plotted by R package “rms”. All statistical analysis was conducted in R software (R 3.5.3, https://www.r-project.org/). Significant level of *P* value was set as < 0.05.

## Results

### Aberrant expression level of DNA repair genes involves in tumorigenesis of colon cancer

**Figure 1:**
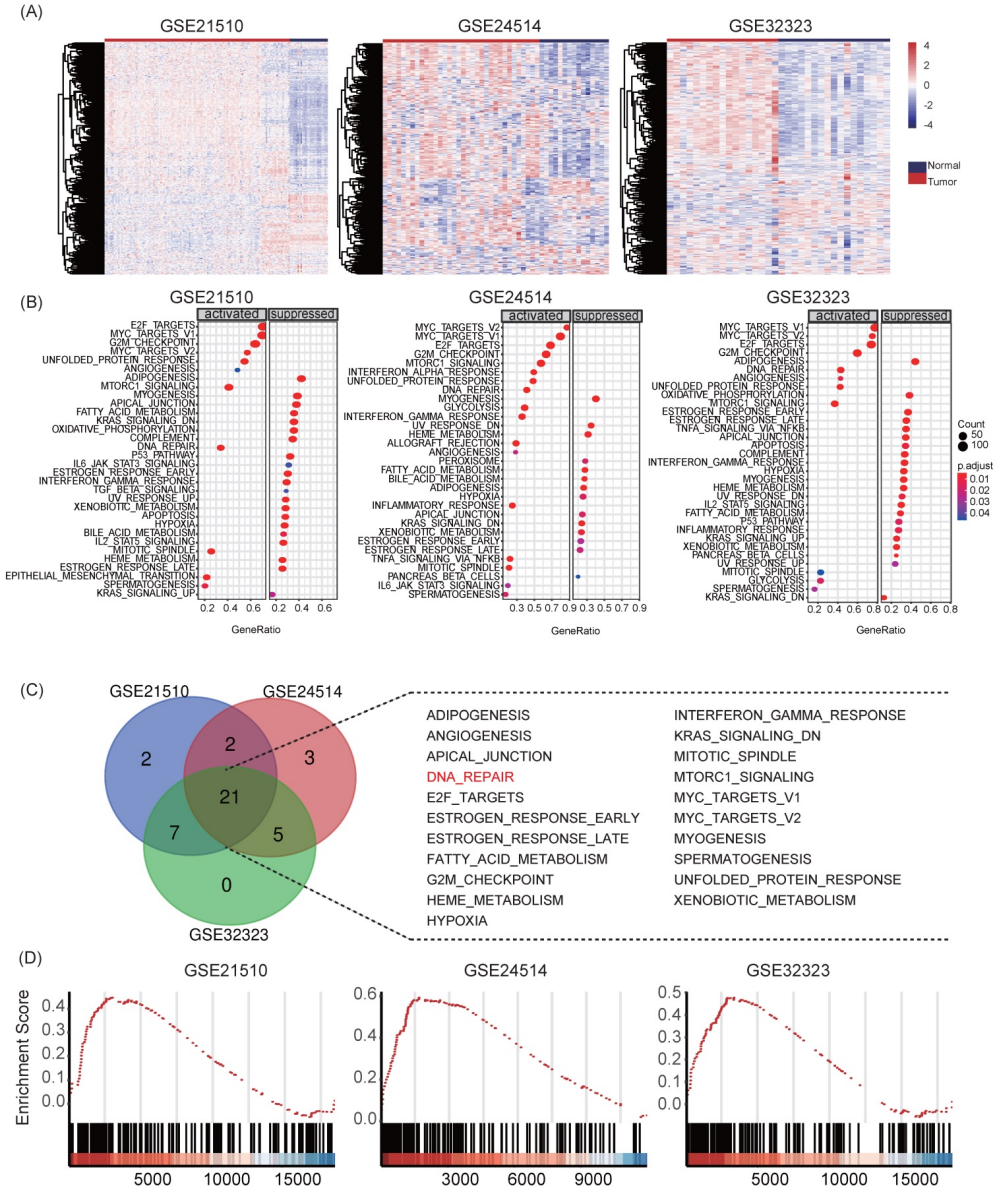
The DNA repair signaling pathway is up-regulated in development in colon cancer. **(A)** Heatmaps of GSE21510, GSE24514 and GSE32323 data sets. Different patterns of transcriptional expression were observed in tumor and non-tumor samples. **(B-D)** Enriched pathways of GSEA, HALLMARK_DNA_REPAIR was activated in all three data sets.

First, we collected transcriptome sequencing data of 231 human tissue samples from three independent GEO datasets (GSE21510, GSE24514 and GSE32323). Heat maps showed that DNA repair genes displayed different expression patterns in normal colon mucosa and colon cancer samples (**Figure 1A**). Moreover, we performed GSEA for hallmarks gene sets in the three datasets. Reports demonstrated that DNA repair pathways were significantly activated in all datasets during the transformation from normal mucosa to colon cancer (**Figure 1B-C**). The normalized enrichment score (NES) of HALLMARK_DNA_REPAIR was 1.7158 (adjust *P* = 0.0052) in GSE21510, 2.2097 (adjust *P* = 0.0051) in GSE24514, and 1.7122 (adjust *P* = 0.0058) in GSE32323, respectively (**Figure 1D**). The results suggested that DRGs play important roles in tumorigenesis in colon cancer and the recognition of key prognostic DRGs might be of great significance in understanding tumor pathological physiology.

### Identification of survival-related DRGs and establishment of the eleven-gene prognostic signature TCGA-COAD cohort

A total of 420 patients diagnosed as colon adenocarcinoma from TCGA-COAD data set was enrolled for establishment of the DRG signature, and 2/3 of them (n = 295) were randomly assigned to the training set. The baseline information of the patients in the training and internal validation cohort was listed in **Table S3-S4**. LASSO Cox regression was conducted to screen survival-related DRGs to predict OS and calculated the risk score (**Figure S1**). An eleven-DRGs‐based prognosis risk score was established, among which six risk and five protective genes were identified for indicating clinical outcomes. The formula of risk factor was as follow:

Risk Score = ACTR8 * (-0.23337) + DMC1 * (-0.16365) + MAGEF1 * 0.042617 + MC1R * 0.108694 + POLG * 0.05497 + RBM17 * 0.418946 + SFPQ * (-0.22302) + TERF2IP * 0.135403 + TP53BP1 * 0.092702 + UIMC1 * (-0.20938) + USP7 * (-0.04848)

**Figure 2:**
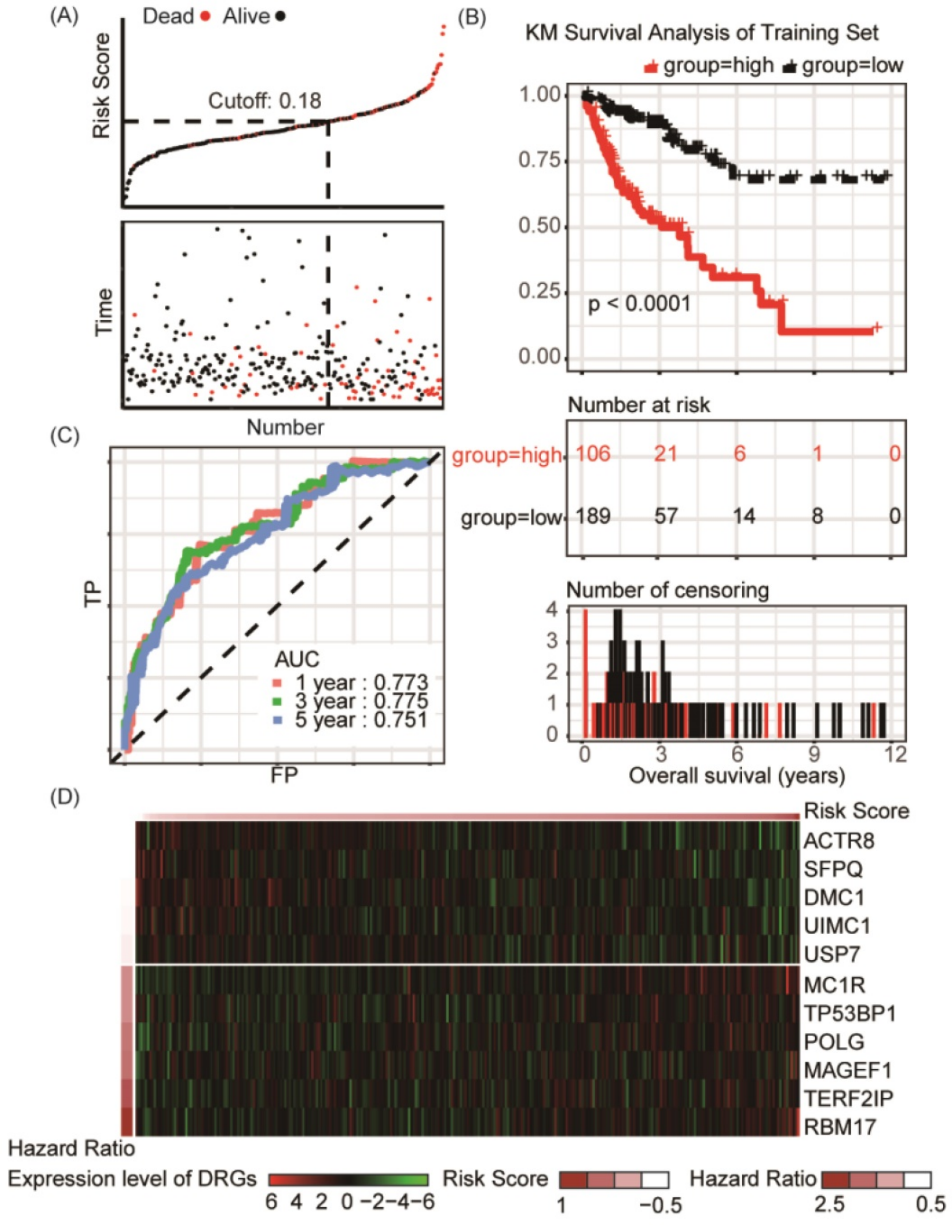
Risk group identified by the DRG classifier, KM survival analysis and ROC curve of TCGA-COAD training set. **(A)** The relationship between alive/dead status with Risk Score and survival time (years). The cutoff of Risk Score was set at 0.18. **(B)** KM survival analysis of overall survival for high-risk or low-risk group patients.** (C)** ROC analysis of the eleven-DRG prognostic signature. The AUC for 1-year, 3-year, 5-year predicting were 0.773, 0.775 and 0.751, respectively. **(D)** Heatmap displayed the expression level of eleven DRGs.

According to the risk factor, patients could be divided into the high risk and the low risk groups, and the cutoff value, obtained by utilizing the assessment of Youden's index, was set at 0.18 for best prediction accuracy (**Figure 2A**). In the training set, Kaplan-Meier survival analysis suggested prognosis of patient in the low risk groups is significantly better than those in high risk groups (*P* < 0.001, **Figure 2B**). ROC curve displayed that the accuracy of the prognostic DRG signature for 1-year, 3-year and 5-year survival was 0.773, 0.775 and 0.751, respectively (**Figure 2C**). Distribution of the risk score, hazard ratio and gene expression of the 11 DRGs in the training dataset were showed in **Figure 2D**. Moreover, analysis of the correlation of risk stratification with clinicopathological data for patients with colon cancer showed that pathological T, pathological N and TNM stage were significant associated with risk stratification in the training set (**Table 1**).

### Internal validation of 11-DRG signature in TCGA-COAD cohort

**Figure 3:**
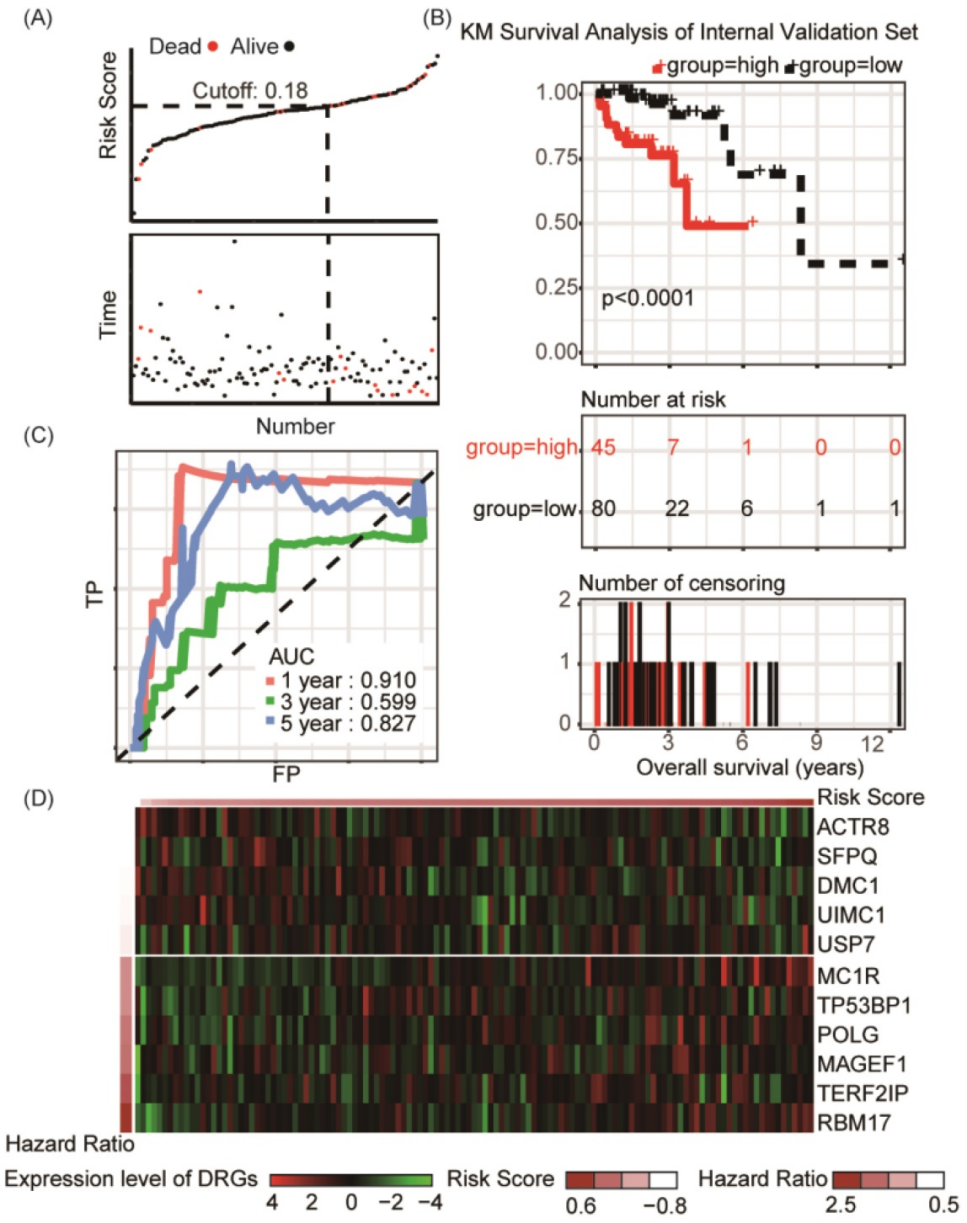
Risk group identified by the DRG classifier, KM survival analysis and ROC curve of TCGA-COAD internal validation set. **(A)** KM survival analysis of overall survival for high-risk or low-risk group patients. **(B)** The relationship between alive/dead status with Risk Score and survival time (years). The cutoff of Risk Score was set at 0.18. **(C)** ROC analysis of the eleven-DRG prognostic signature. The AUC for 1-year, 3-year, 5-year predicting were 0.910, 0.599 and 0.827, respectively. (D) Heatmap displayed the expression level of eleven DRGs.

**Figure 4:**
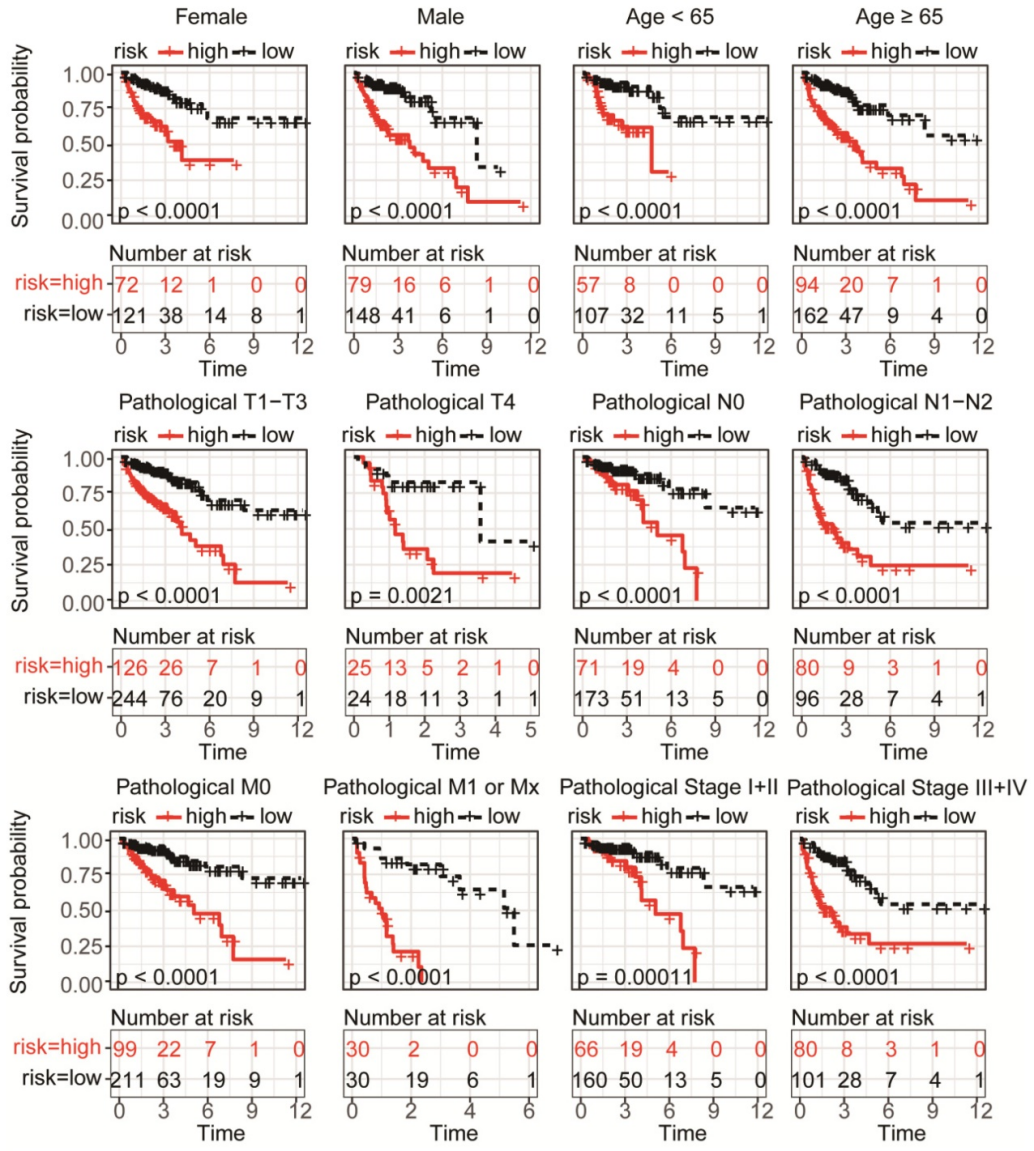
Subgroup KM analysis in high or low risk group patients of TCGA-COAD according to clinical characteristics. Significance differences of overall survival was detected in all subgroup analysis, including distinct gender, age, pathological T, N, M and stage.

To validate the predictive capability of the DRG signature as a prognostic indictor, the rest 125 samples of TCGA-COAD cohort were used for internal validation, which showed significant differences of overall survival between distinct risk groups (P < 0.0001, **Figure 3A-B**). Result of ROC analysis showed the AUC for OS prediction was 0.910 for 1-year, 0.599 for 3- year and 0.827 for 5-year in the internal validation set, respectively (**Figure 3C**). Distribution of the risk score, hazard ratio and gene expression of the 11 DRGs in the internal validation dataset were showed in **Figure 3D**. However, there was no significant difference between clinical characteristics and risk stratification in the internal validation set (**Table 1**). Distribution of various clinical parameters at low or high risk group was plotted in **Figure S2**. Furthermore, the stratified analyses of patients with different clinicopatholoical characteristics of the whole TCGA-COAD cohort showed that OS of the patients could be significantly distinguished in all subgroups (**Figure 4**).

### External validation of 11-DRG signature in independent GEO colon cancer cohort

**Figure 5:**
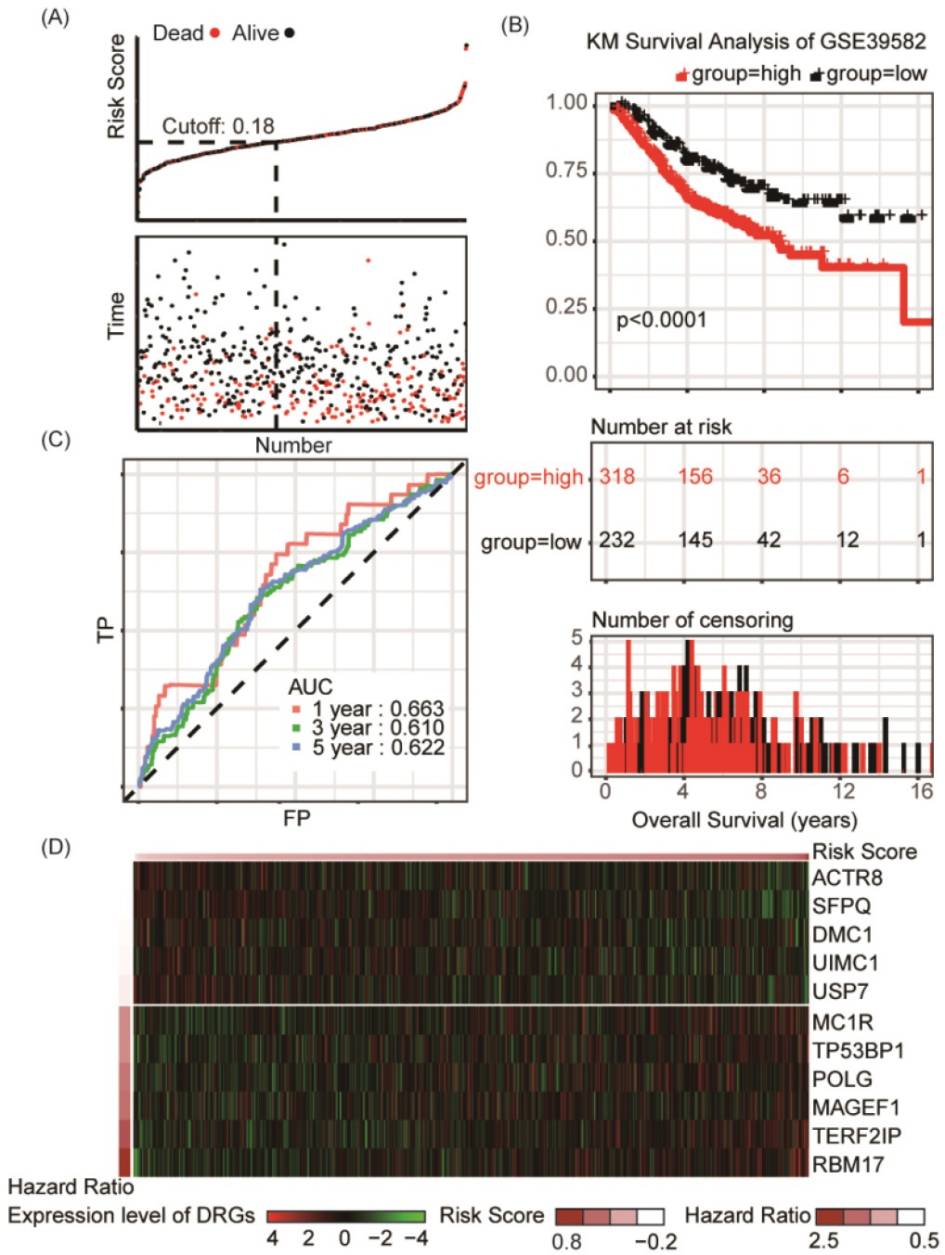
Risk group identified by the DRG classifier, KM survival analysis and ROC curve of GSE39582 dataset. **(A)** The relationship between alive/dead status with Risk Score and survival time (years). The cutoff of Risk Score is set at 0.18. **(B)** KM survival analysis of overall survival for high-risk or low-risk group patients. **(C)** ROC analysis of the eleven-DRG prognostic signature. The AUC for 1-year, 3-year, 5-year predicting were 0.663, 0.610 and 0.622, respectively. **(D)** Heatmap displayed the expression level of eleven DRGs.

Another GEO dataset (GSE39582), which containing 550 patients with definite diagnosis of colon cancer, was served as the external validation set (**Table S5**). Significant differences of overall survival between distinct risk groups were observed in external validation set (*P* < 0.001, **Figure 5A-B**). In dataset GSE39582, the AUC was 0.663, 0.610 and 0.622 for 1-year, 3-year, 5-year survival, respectively (**Figure 5C**). Distribution of the risk score, hazard ratio and gene expression of the 11 DRGs in the external validation dataset were showed in **Figure 5D**. Moreover, analysis of the correlation of risk stratification with clinicopathological data for patients with colon cancer also showed that pathological T, pathological N and TNM stage were significant associated with risk stratification in the external validation set (**Table 1**). The stratified analyses of patients with different clinicopatholoical characteristics from the training set showed that OS of the patients could be significantly distinguished in most of subgroups except patients younger than 65-year-old, at T4 stage, N1-2 stage, or M1-x stage (**Figure S3**).

### Evaluation of independent prognostic factors in colon cancer

**Figure 6:**
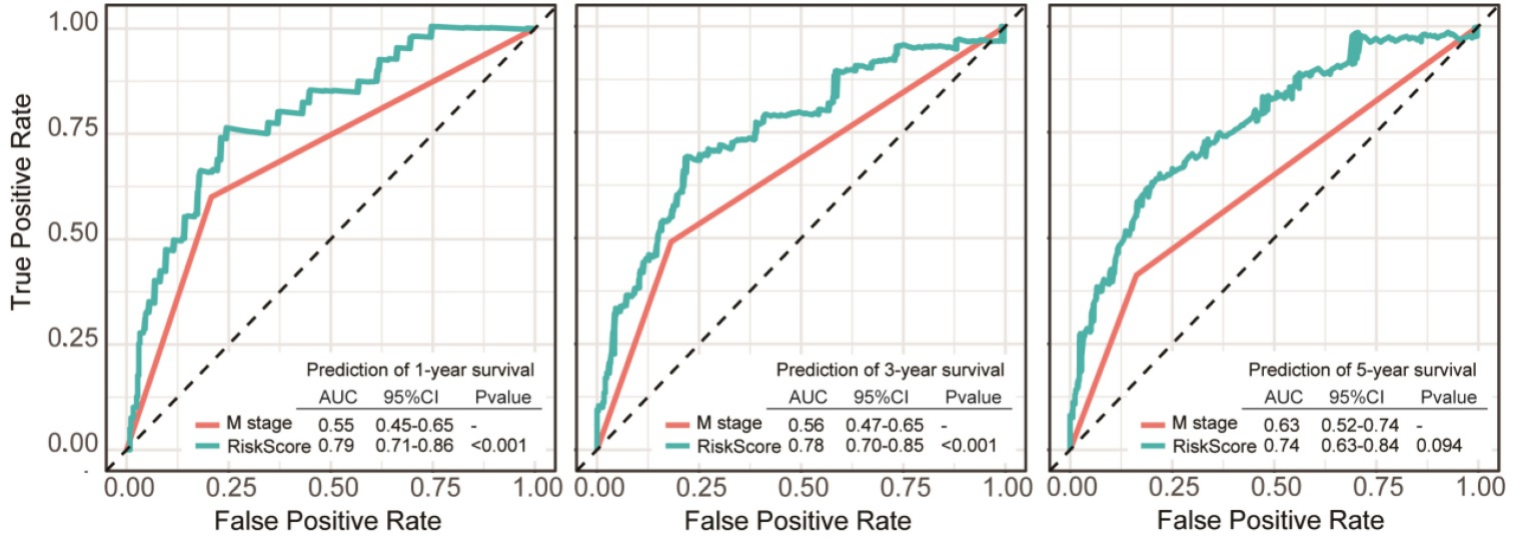
Comparisons of sensitivity and specificity for survival prediction by DRG signature and pathologic M stage as independent factors. The eleven-DRG signature showed a better capability for survival prediction than pathologic M stage. Significant differences reached at 1-year and 3-year prediction.

Prognostic factors of overall survival for colon cancer in the TCGA-COAD set were identified using univariate and multivariate Cox regression analyses. The results revealed that Risk Score and pathologic M stage were independent indicators (**Table 2**). This suggested that the 11-DRG signature has good independence in clinical application. In ROC curve comparing multi-factors to predict survival probability of 1 year, 3 years and 5 years, the DRG signature showed better capability of prognostic prediction than pathologic M stage in 1-year and 3-year OS (**Figure 6**).

**Figure 7:**
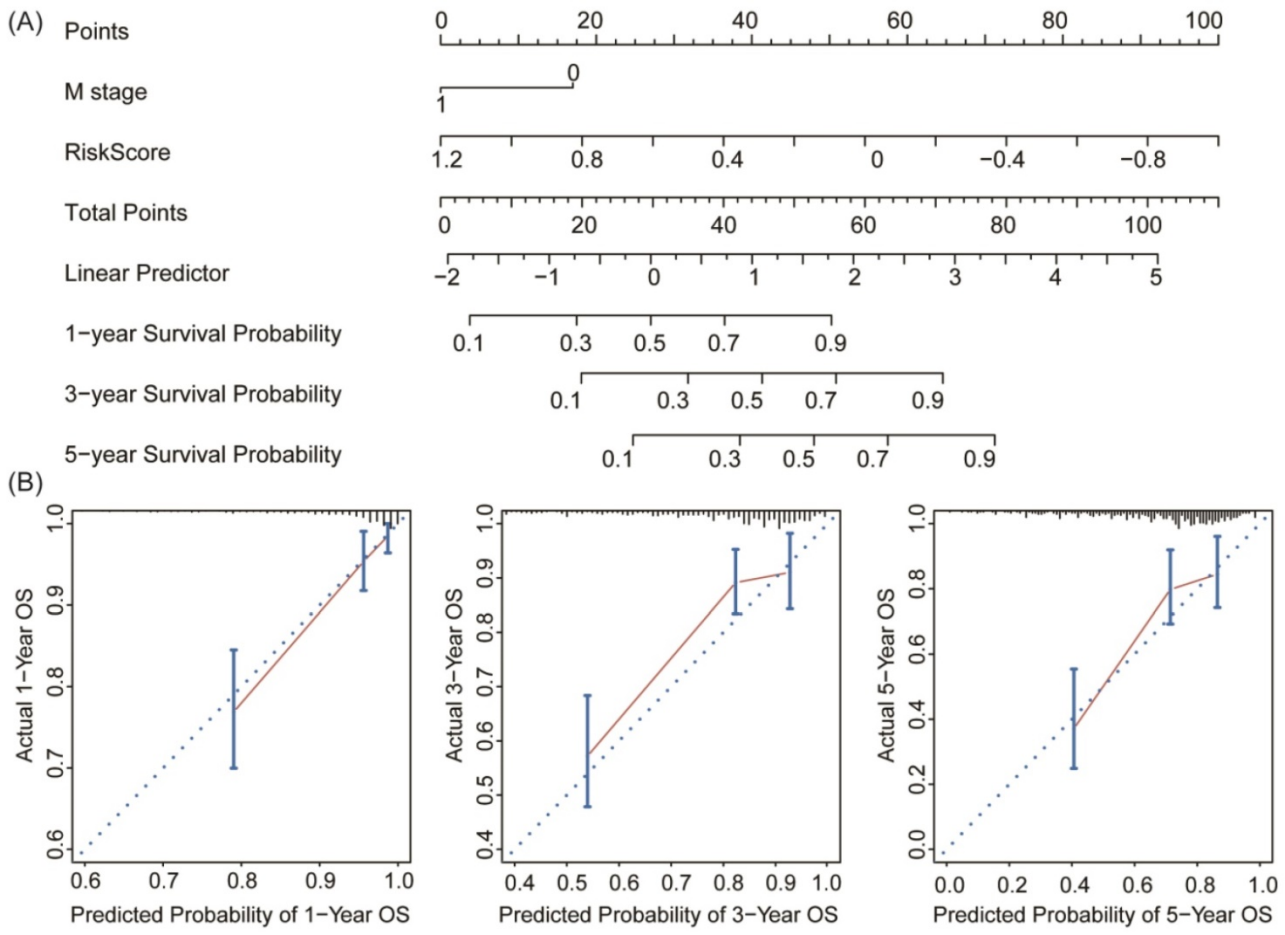
Nomogram and calibration analysis for the DRG prognostic signature. **(A)** Nomogram plotted by the independent factors of patients' survival. The probability of long-term survival can be calculated by adding the corresponding points of M stage and Risk Score in the nomogram. **(B)** Calibration plots displayed the relationship between actual and the nomogram-predicted survival, which indicated a powerful predicting capability of the nomogram.

To establish a clinically applicable method for predicting the survival probability of patients with colon cancer, we developed a nomogram to predict the probability of the 1‐, 3‐ and 5‐year OS in the whole TCGA-COAD cohort. The predictors of the nomogram included both independent prognostic factors (pathologic M stage and 11-DRG signature; **Table 2**). Finally, we drew a nomogram for predicting the survival probability of patients considering the feasibility of clinical practice (**Figure 7A**). Calibration plots demonstrated stability of the nomogram in predicting 3- or 5- year overall survival (**Figure 7B**). These findings demonstrated that the nomogram is a robust model for predicting survival for patients with colon cancer, which might facilitate patient counselling, decision‐making and follow‐up scheduling.

## Discussion

Increasing evidences has elucidated the roles of DNA damage and repair deficiencies in malignancies, including colon cancer 7. Different expression patterns of DRGs are frequently detected in normal mucosa and cancer tissues, which were closely related to patients' prognosis 17, 18. However, large variations in the mechanisms of tumorigenesis and heterogeneity of tumors suggest the obviously limited prognostic value of individual gene detection. Establish a multi-gene prognostic panel instead of a single gene biomarker provides potentially more optimal feasibility in predicting clinical outcome for patients with colon cancer 19, 20. Up to now, in colon cancer, no applicable research on transcriptional patterns of DRGs and its prospective prognostic value has been reported. Thus, we developed an 11-DRG signature predicting the survival outcomes of patients with colon cancer. In the present study, by screened the candidate prognostic signature in TCGA and GEO colon cancer datasets, a 11-DRG signature was identified to be able to stratified the survival risk of patients with colon cancer, and the overall survival of high-risk group was significantly worse than that of low-risk group. The signature also performed well in internal and external validation cohorts. Univariate and multivariate Cox regression determined that Risk Score was an independent prognosis factor for OS in patients with colon cancer. Furthermore, a nomogram was developed and validated to predict OS based on the age of patients. The nomogram provided favorable discrimination and calibration plots. The results of this nomogram may help to optimize the preoperative management of patients with colon cancer.

In the current study, transcriptome sequencing data and clinical information was obtained from public database, TCGA and GEO. To confirm the involvement of DRGs in development of colon cancer, we conducted analysis of expression profiles for patients from three GEO datasets, discovering higher transcriptional activity of DRGs in the most neoplastic epithelium. Furthermore, all of the datasets dramatically achieved “HALLMARK_DNA_REPAIR” enrichment in GSEA, which suggested that the abundance of DRGs might be a candidate indicator of malignant transformation proceedings in colon cancer and, to some extent, high levels of DRGs expression might be protective factors for prognostic prediction. Given the fact above, we established a novel prognostic DRG signature consist of 11 genes, which was validated as an independent predictor of patient's survival for colon cancer. The Risk Score assessment could successfully classify the patients into high or low risk group with significant differences in overall survival. In addition, further validation was conducted in an internal data set and an independent external set, which reflected the good accuracy and reproducibility of the modal.

In our study, we identified 11 DRGs (ACTR8, DMC1, MAGEF1, MC1R, POLG, RBM17, SFPQ, TERF2IP, TP53BP1, UIMC1, USP7), whose altered expression level was closely related to the prognosis of colon cancer patients. TP53BP1, also known as 53BP1, encodes a kind of chromatin-binding protein, which is a key component in DNA double-strand break signaling in response to DNA damage by promoting non-homologous and joining mediated repair 21, 22. The functions of TP53BP1 in chromatin stability determines its critical role in cancer and reports emergence in breast, lung, prostate and colorectal cancer 23-27. Bi *et al.* 28 demonstrated that deficiency of TP53BP1 significantly inhibited apoptosis of tumor cells and prompted proliferation and S phase accumulation in cell cycle. UIMC1 (also known as RAP80) is a member of BRCA1-A complex, and engages in checkpoint arrest in cell cycle and was lately recognized as a regulator in tumor cell apoptosis and epithelium mesenchymal transition 29, 30. SFPQ (also named as PSF) translates protein binding to a nuclear receptor PPARγ and modulate growth of colon cancer cells 31. Although TP53BP1 and UIMC1 have been identified as tumor suppressor 28-30 and SFPQ has been identified as oncogene 31, TP53BP1 was ranked as risk factor, while UIMC1 and SFPQ are protect factor in our 11-gene signature, which revealed the complication of molecular signaling, specific gene may play totally opposite role during tumorigenesis and development. This phenomenon is an important research point and needs for further more attention. Besides, roles of some of the candidate genes that play in colon cancer have not been revealed. However, our analysis suggested these DRGs might provide promising value in colon cancer progression.

In conclusion, the current study proposed a newly developed eleven-DRG signature as a practical prognostic predictor for patients with colon cancer, which can contribute independent value in identifying clinical outcomes that complements the TNM system in colon cancer.

## Supplementary Material

Supplementary figures and tables.

## Figures and Tables

**Table 1:** Associations with risk group and clinical characteristics in the training and validation sets

	TCGA-COAD training set			TCGA-COAD validation set			GSE39582		
	High risk	Low risk	*P*	High risk	Low risk	*P*	High risk	Low risk	*P*
**Gender**			0.929			0.265			0.957
Female	48	88		24	33		142	105	
Male	58	101		21	47		176	127	
**Age**	0.979					0.744			0.988
<65	40	73		17	34		122	88	
≥65	66	116		28	46		196	144	
**T stage**			**0.000**			0.805			**0.000**
T1	3	3		2	3		1	14	
T2	7	46		7	13		23	20	
T3	76	121		31	59		207	148	
T4	20	19		5	5		75	42	
**N stage**			**0.001**			0.179			**0.016**
N0	46	124		25	49		153	139	
N1	32	41		8	20		83	51	
N2	28	24		12	11		70	34	
**M stage**	0.070					0.173			0.368
M0	69	150		30	61		266	203	
M1	20	22		10	8		38	21	
Mx	13	16		5	11		2	0	
**Stage**			**0.001**			0.321			**0.003**
I	7	42		7	14		12	24	
II	37	74		15	32		140	116	
III	38	47		12	24		129	71	
IV	20	22		10	8		37	21	

**Table 2:** Univariate and multivariate Cox analysis of Risk Score and clinical characteristics on overall survival in TCGA-COAD

Variables	Univariate analysis		Multivariate analysis	
	HR (95% CI)	*P* value	HR (95% CI)	*P* value
Age	1.02 (1.00-1.04)	0.064		
Gender	1.19 (0.79-1.81)	0.405		
Stage	2.40 (1.87-3.09)	<0.001	1.58 (0.63-0.99)	0.058
T	2.95 (1.95-4.46)	<0.001	1.42 (0.76-2.64)	0.400
N	2.16 (1.69-2.76)	<0.001	0.66 (0.26-1.71)	0.606
M	3.80 (2.48-5.83)	<0.001	2.37 (1.43-3.90)	0.047
Risk Score	24.27 (11.21-52.55)	<0.001	14.65 (6.53-32.83)	<0.001
