# Supplementary material for: Development and validation of a DNA repair gene signature for prognosis prediction in Colon Cancer
Source: J Cancer. 2020 Aug 12;11(20):5918–28. doi: 10.7150/jca.46328 (PMC7477412; doi:10.7150/jca.46328)
Supplement: Supplementary file 1 — Supplementary figures and tables. [file jcav11p5918s1.pdf]

Table S1 Summary of GEO datasets included

GEO dataset	Platform	Enrolled sample	
		Tumor	Non-tumor
GSE21510	Affymetrix Human Genome U133 Plus 2.0 Array	123	25
GSE24514	Affymetrix Human Genome U133A Array	34	15
GSE32323	Affymetrix Human Genome U133 Plus 2.0 Array	17	17
GSE39582	Affymetrix Human Genome U133 Plus 2.0 Array	550	0

Table S2 DNA repair genes list

Gene symbols of DNA repair genes	(n = 476)
ABL1, ACTL6A, ACTR2, ACTR5, ACTR8, ADPRHL2, ALKBH1, ALKBH2, ALKBH3, ALKBH5, ANKLE1, AP5S1, APBB1, APEX1, APEX2, APLF, APTX, AQR, ASCC1, ASCC2, ASCC3, ASF1A, ASTE1, ATM, ATR, ATRX, ATXN3, AUNIP, AXIN2, BABAM1, BACH1, BARD1, BCCIP, BLM, BOD1L1, BRCA1, BRCA2, BRCC3, BRIP1, BTG2, C14orf39, CBX8, CCDC155, CCNH, CDC14B, CDC45, CDC5L, CDC7, CDCA5, CDK1, CDK2, CDK7, CDK9, CDKN2D, CEBPG, CEP164, CETN1, CETN2, CHAF1A, CHAF1B, CHCHD4, CHD1L, CHEK1, CHEK2, CHRNA4, CIB1, CINP, CLSPN, COPS2, COPS3, COPS4, COPS5, COPS6, COPS7A, COPS7B, COPS8, CSNK1E, CUL4A, CUL4B, DCLRE1A, DCLRE1B, DCLRE1C, DDB1, DDB2, DDX1, DDX11, DEK, DHX9, DMAP1, DMC1, DNA2, DNTT, DTL, DTX3L, EEPD1, EGFR, EID3, EME1, EME2, ENDOV, EP300, EPC2, ERCC1, ERCC2, ERCC3, ERCC4, ERCC5, ERCC6, ERCC6L2, ERCC8, ESCO2, ETAA1, EXD2, EXO1, EXO5, EYA1, EYA2, EYA3, EYA4, FAM168A, FAN1, FANCA, FANCB, FANCC, FANCD2, FANCE, FANCF, FANCG, FANCI, FANCL, FANCM, FBXO6, FEN1, FGF10, FIGN, FIGNL1, FMN2, FOXM1, FTO, FUS, FZR1, GADD45A, GEN1, GGN, GINS2, GINS4, GPS1, GTF2H1, GTF2H3, GTF2H4, GTF2H5, H2AFX, HDAC10, HELB, HELQ, HERC2, HINFP, HIST1H4A, HIST1H4D, HIST1H4E, HIST1H4H, HIST1H4I, HIST1H4J, HIST1H4L, HIST3H2A, HIST3H3, HMGA1, HMGA2, HMGB1, HMGB2, HMGN1, HSF1, HUS1, HUS1B, HUWE1, IGHMBP2, INIP, INO80, INO80B, INO80C, INO80D, INO80E, INTS3, ISG15, ISY1, JMY, KAT5, KDM1A, KDM2A, KDM4D, KIF22, KIN, KLHL15, LIG1, LIG3, LIG4, MAD2L2, MAGEF1, MBD4, MC1R, MCM8, MCM9, MCMDC2, MCRS1, MDC1, MEN1, MGME1, MGMT, MLH1, MLH3, MMS19, MNAT1, MORF4L1, MORF4L2, MPG, MSH2, MSH3, MSH4, MSH6, MTA1, MUS81, MUTYH, NABP1, NABP2, NBN, NCOA6, NEIL2, NEIL3, NFRKB, NIPBL, NONO, NPAS2, NPLOC4, NPM1, NSMCE1, NSMCE2, NSMCE4A, NTHL1, NUCKS1, NUDT1, NUDT16,	

NUDT16L1, OGG1, PAGR1, PALB2, PARG, PARK7, PARP1, PARP10, PARP2, PARP3, PARP4, PARP9, PARPBP, PAXIP1, PCNA, PDS5A, PDS5B, PIAS4, PIF1, PML, PMS1, PNKP, POLA1, POLB, POLD1, POLD2, POLD3, POLD4, POLE, POLE2, POLG, POLG2, POLH, POLI, POLK, POLL, POLM, POLN, POLQ, POLR2A, POLR2B, POLR2C, POLR2D, POLR2E, POLR2F, POLR2G, POLR2H, POLR2I, POLR2J, POLR2K, POLR2L, PPIE, PPP4C, PPP4R2, PPP5C, PRIMPOL, PRKCG, PRKDC, PRMT6, PRPF19, PSMD14, PSME4, PTTG1, RAD1, RAD17, RAD18, RAD21, RAD21L1, RAD23A, RAD23B, RAD50, RAD51, RAD51AP1, RAD51B, RAD51C, RAD51D, RAD52, RAD54B, RAD54L, RAD9A, RAD9B, RBBP8, RBM14, RBM17, RBX1, RCHY1, REC8, RECQL, RECQL4, RECQL5, REV1, REV3L, REXO4, RFC1, RFC2, RFC3, RFC4, RFC5, RFWD3, RHNO1, RMI1, RMI2, RNASEH2A, RNF111, RNF113A, RNF138, RNF168, RNF169, RNF8, RPA1, RPA2, RPA3, RPA4, RPAIN, RPS27A, RPS3, RRM2B, RTEL1, RUVBL1, RUVBL2, SAMHD1, SETD2, SETMAR, SETX, SFPQ, SFR1, SHPRH, SIRT1, SLC30A9, SLX4, SMARCAD1, SMARCAL1, SMC1A, SMC3, SMC5, SMC6, SMCHD1, SMUG1, SPATA22, SPIDR, SPIRE1, SPIRE2, SPO11, SPRTN, SSRP1, STUB1, SUMO1, SUPT16H, SWI5, SWSAP1, SYCP1, TAOK1, TAOK3, TCEA1, TDG, TDP1, TDP2, TERF2, TERF2IP, TEX12, TEX15, TFIP11, TFPT, TICRR, TIMELESS, TMEM161A, TNKS1BP1, TNP1, TONSL, TOPBP1, TP53, TP53BP1, TP73, TRIM25, TRIM28, TRIP12, TRIP13, TRRAP, TTC5, TWIST1, UBA52, UBB, UBC, UBE2A, UBE2B, UBE2D3, UBE2L6, UBE2N, UBE2T, UBE2U, UBE2V2, UBE2W, UBR5, UCHL5, UHRF1, UIMC1, UNG, UPF1, USP1, USP10, USP28, USP3, USP43, USP45, USP47, USP51, USP7, UVRAG, UVSSA, VCP, WAS, WDHD1, WDR48, WDR70, WRAP53, WRN, WRNIP1, XAB2, XPA, XPC, XRCC1, XRCC2, XRCC3, XRCC4, XRCC5, XRCC6, YY1, ZBTB1, ZBTB7A, ZFYVE26, ZMPSTE24, ZNF365, ZNF830, ZRANB3, ZSWIM7

Table S3 Clinicopathological characteristics in TCGA-COAD training cohort (N = 295)

	Alive (N = 221)		Dead (N = 74)		Total (N = 295)	
Age						
<65	91	(41.20%)	22	(29.70%)	113	(38.30%)
>=65	130	(58.80%)	52	(70.30%)	182	(61.70%)
Gender						
female	104	(47.10%)	32	(43.20%)	136	(46.10%)
male	117	(52.90%)	42	(56.80%)	159	(53.90%)
Pathological T stage						
T1	5	(2.30%)	1	(1.40%)	6	(2.00%)
T2	49	(22.20%)	4	(5.40%)	53	(18.00%)
T3	147	(66.50%)	50	(67.60%)	197	(66.80%)
T4	20	(9.00%)	19	(25.70%)	39	(13.20%)
Pathological N stage						
N0	145	(65.60%)	25	(33.80%)	170	(57.60%)
N1	50	(22.60%)	23	(31.10%)	73	(24.70%)
N2	26	(11.80%)	26	(35.10%)	52	(17.60%)
Pathological M stage						
M0	178	(81.70%)	41	(56.90%)	219	(75.50%)
M1	20	(9.20%)	22	(30.60%)	42	(14.50%)
Mx	20	(9.20%)	9	(12.50%)	29	(10.00%)
Pathological Stage						
Stage I	46	(21.20%)	3	(4.30%)	49	(17.10%)
Stage II	92	(42.40%)	19	(27.10%)	111	(38.70%)
Stage III	59	(27.20%)	26	(37.10%)	85	(29.60%)
Stage IV	20	(9.20%)	22	(31.40%)	42	(14.60%)

Table S4 Clinicopathological characteristics in TCGA-COAD validation cohort (N = 125)

	Alive (N = 108)	Dead (N = 17)	Total (N = 125)
Age			
<65	45 (41.7%)	6 (35.3%)	51 (40.8%)
>=65	63 (58.3%)	11 (64.7%)	74 (59.2%)
Gender			
female	50 (46.3%)	7 (41.2%)	57 (45.6%)
male	58 (53.7%)	10 (58.8%)	68 (54.4%)
Pathological T stage			
T1	5 (4.6%)	0 (0.0%)	5 (4.0%)
T2	20 (18.5%)	0 (0.0%)	20 (16.0%)
T3	75 (69.4%)	15 (88.2%)	90 (72.0%)
T4	8 (7.4%)	2 (11.8%)	10 (8.0%)
Pathological N stage			
N0	67 (62.0%)	7 (41.2%)	74 (59.2%)
N1	27 (25.0%)	1 (5.9%)	28 (22.4%)
N2	14 (13.0%)	9 (52.9%)	23 (18.4%)
Pathological M stage			
M0	86 (79.6%)	5 (29.4%)	91 (72.8%)
M1	9 (8.3%)	9 (52.9%)	18 (14.4%)
Mx	13 (12.0%)	3 (17.6%)	16 (12.8%)
Pathological Stage			
Stage I	21 (19.8%)	0 (0.0%)	21 (17.2%)
Stage II	43 (40.6%)	4 (25.0%)	47 (38.5%)
Stage III	33 (31.1%)	3 (18.8%)	36 (29.5%)
Stage IV	9 (8.5%)	9 (56.3%)	18 (14.8%)

Table S5 Clinicopathological characteristics in GSE39582 validation cohort (N = 550)

	Alive (N = 365)		Dead (N = 185)		Total (N = 550)	
Age						
<65	152	(41.6%)	58	(31.4%)	210	(38.2%)
>=65	213	(58.4%)	127	(68.6%)	340	(61.8%)
Gender						
female	174	(47.7%)	73	(39.5%)	247	(44.9%)
male	191	(52.3%)	112	(60.5%)	303	(55.1%)
Pathological T stage						
T1	13	(3.6%)	2	(1.2%)	15	(2.8%)
T2	35	(9.8%)	8	(4.6%)	43	(8.1%)
T3	246	(68.9%)	109	(63.0%)	355	(67.0%)
T4	63	(17.6%)	54	(31.2%)	117	(22.1%)
Pathological N stage						
N0	213	(59.7%)	79	(45.7%)	292	(55.1%)
N1	89	(24.9%)	45	(26.0%)	134	(25.3%)
N2	55	(15.4%)	49	(28.3%)	104	(19.6%)
Pathological M stage						
M0	336	(94.1%)	133	(76.9%)	469	(88.5%)
M1	20	(5.6%)	39	(22.5%)	59	(11.1%)
Mx	1	(0.3%)	1	(0.6%)	2	(0.4%)
Pathological Stage						
Stage I	30	(8.2%)	6	(3.2%)	36	(6.5%)
Stage II	182	(49.9%)	74	(40.0%)	256	(46.5%)
Stage III	133	(36.4%)	67	(36.2%)	200	(36.4%)
Stage IV	20	(5.5%)	38	(20.5%)	58	(10.5%)

Supplementary figure legends

Figure S1. Establishment of the DRGs prognostic model with LASSO penalty.
The top figure showed optimal penalty parameter λ (lambda) chose by cross-validation method was 0.058. The bottom figure exhibited the gene selected in the LASSO penalty with specific lambda value.

Figure S2. Distribution of clinical parameters in high risk or low risk group.
Color distinguished different levels of clinical pathological characteristic of patients in high or low group.

Figure S3. Subgroup KM analysis in high or low risk group patients of GSE39582 according to clinical characteristics.
Significance differences of overall survival was detected in most of subgroups except patients younger than 65-year-old, at T4 stage, N1-2 stage, or M1-x stage.

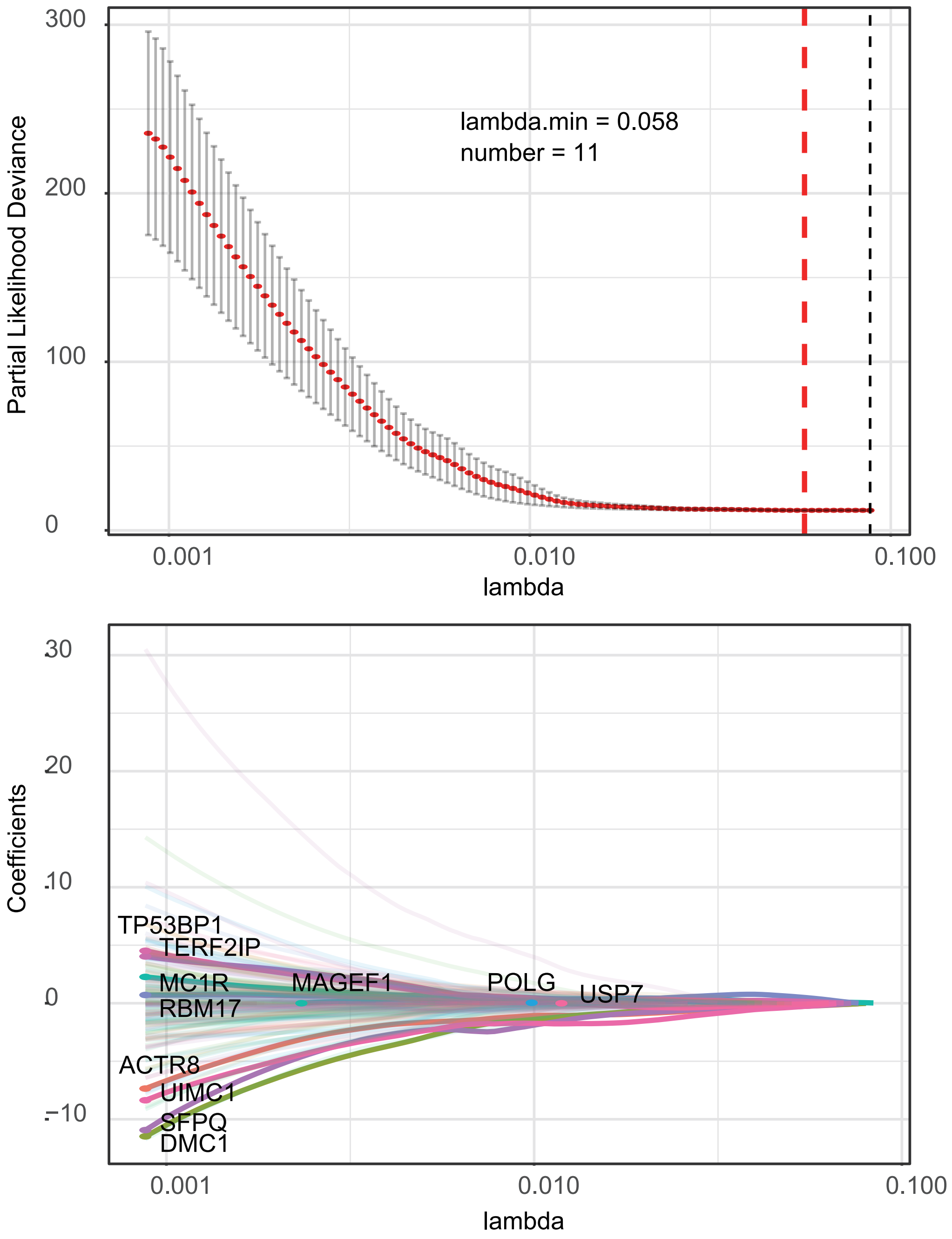
300
200
100
0
Partial Likelihood Deviance
lambda.min = 0.058
number = 11
0.001
0.010
0.100
lambda
30
20
10
0
−10
Coefficients
TP53BP1
TERF2IP
MC1R
RBM17
MAGEF1
POLG
USP7
ACTR8
UIMC1
SFPQ
DMC1
0.001
0.010
0.100
lambda

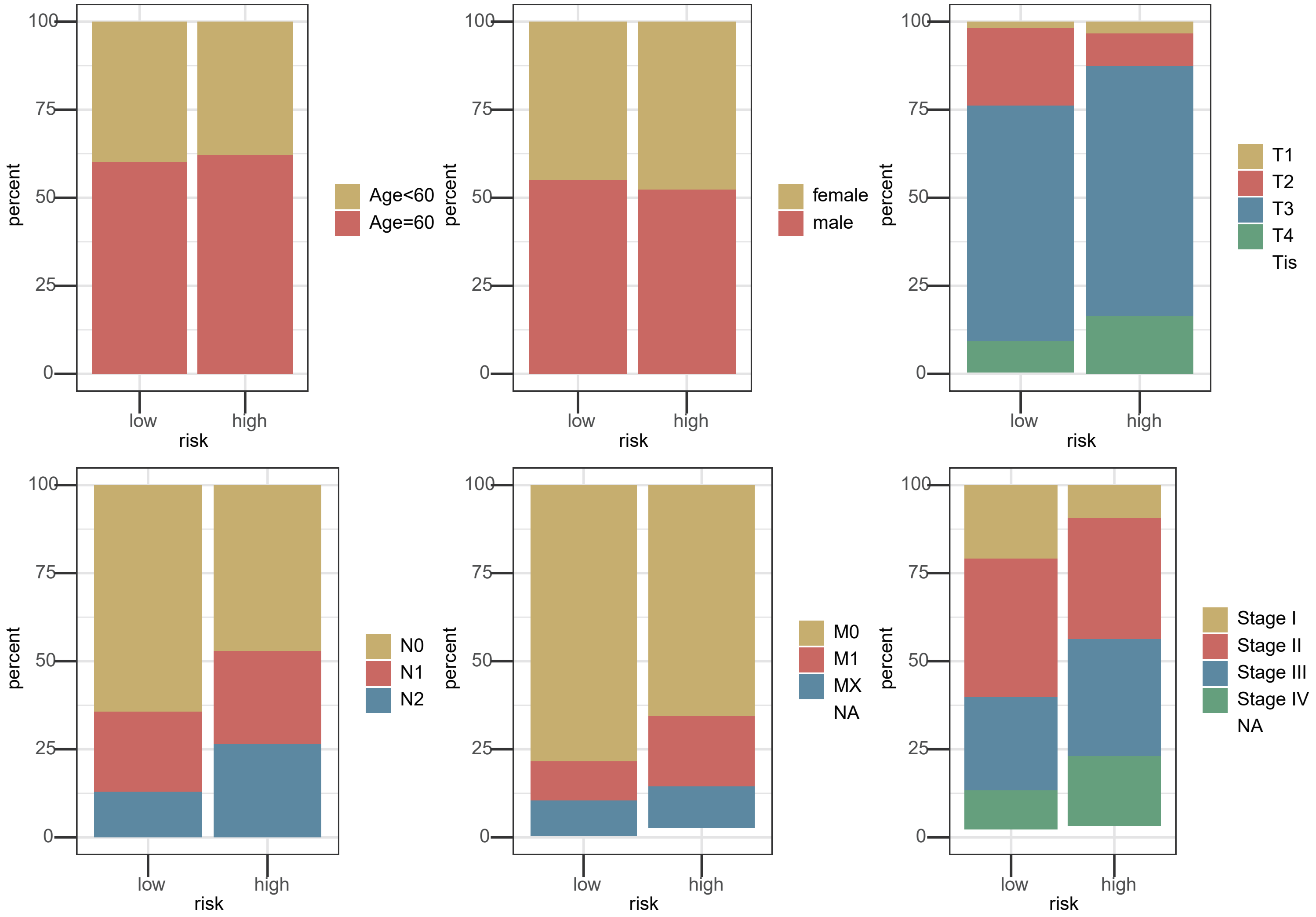
percent
100
75
50
25
0
low
high
risk
Age<60
Age=60
female
male
T1
T2
T3
T4
Tis
N0
N1
N2
M0
M1
MX
NA
Stage I
Stage II
Stage III
Stage IV
NA

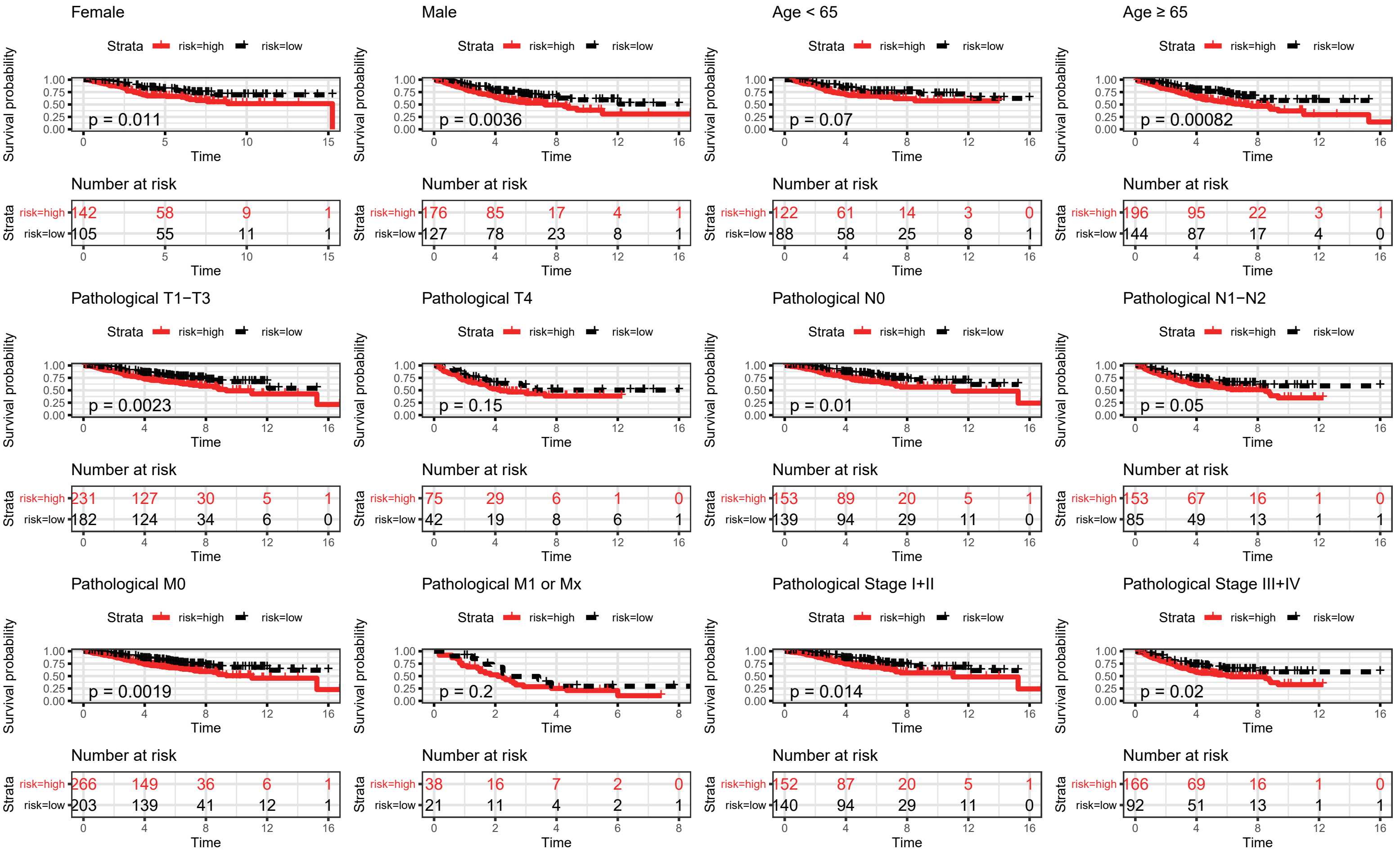
Female
Strata risk=high risk=low
Survival probability
p = 0.011
Time
Number at risk
risk=high 142 58 9 1
risk=low 105 55 11 1
Male
p = 0.0036
risk=high 176 85 17 4 1
risk=low 127 78 23 8 1
Age < 65
p = 0.07
risk=high 122 61 14 3 0
risk=low 88 58 25 8 1
Age ≥ 65
p = 0.00082
risk=high 196 95 22 3 1
risk=low 144 87 17 4 0
Pathological T1−T3
p = 0.0023
risk=high 231 127 30 5 1
risk=low 182 124 34 6 0
Pathological T4
p = 0.15
risk=high 75 29 6 1 0
risk=low 42 19 8 6 1
Pathological N0
p = 0.01
risk=high 153 89 20 5 1
risk=low 139 94 29 11 0
Pathological N1−N2
p = 0.05
risk=high 153 67 16 1 0
risk=low 85 49 13 1 1
Pathological M0
p = 0.0019
risk=high 266 149 36 6 1
risk=low 203 139 41 12 1
Pathological M1 or Mx
p = 0.2
risk=high 38 16 7 2 0
risk=low 21 11 4 2 1
Pathological Stage I+II
p = 0.014
risk=high 152 87 20 5 1
risk=low 140 94 29 11 0
Pathological Stage III+IV
p = 0.02
risk=high 166 69 16 1 0
risk=low 92 51 13 1 1
